# Injurious Effects of Vulcanized Rubber

**Published:** 1877-11

**Authors:** L. P. Haskell


### INJURIOUS EFFECT OF VULCANIZED RUBBER.

BY L. P. HASKELL.

It is strange that among the objectionable features in the use of vulcanized rubber in the mouth, the most objectionable is the least referred to, viz; the *constant absorption* of *alveolus* resulting therefrom.

It cannot be that this effect is overlooked by dentists generally. Can it be that it is ignored for prudential reasons? Doubtless a majority of those who commenced practice since the introduction of rubber are not sufficiently familiar with the use of metal plates to have noticed the difference in their effect. Nevertheless it is true that *vulcanized rubber* (and I think *celluloid* too) *is producing incalculable injury to the mouths of all who wear it.*

Thirty-two years experience, exclusively in mechanical dentistry, has given me ample opportunity to satisfy myself as to the correctness of this statement, and it seems to me that it is fully time that the profession were aroused to the importance of the subject, so that the public were enlightened as to the deleterious effects of the material now so generally used for artificial dentures.

The case is plain and can be readily understood by the patient, and instead of universal recommendation of this material for permanent work, a statement of its nature should be made so that the patient can have a choice in the matter, and not be led to think that it is not only a good material, but really the best for artificial dentures.

As there are many in the profession who do not know the facts in the case, I will state them in a few words. These vegetable bases are *non-conductors of heat*, and it is to the undue retention of heat in the mucous membrane, confined with pressure, that the absorbents are unduly excited, and as a result there is a constant loss of bony tissue. Now for the proof of this assertion, I have never seen a mouth where a rubber plate had been worn five years and upward, but there was manifestly an undue absorption of bone. In the upper jaw, to such an extent, that often there is nothing left but a flabby ridge; in the lower jaw very often a total disappearance of ridge and often a depression.

I do not deny that *sometimes* there is undue absorption when wearing metal plates, but those are the *exceptions* and not the *rule* as in the other case, and arising too from undue pressure, long continued at one point, or to some peculiar idiosyncrasy or constitutional taint.

This fact was emphasized in my own mind more fully upon a recent visit to Boston, where I saw various mouths wearing plates of gold and continuous gum, which I made 22 to 24 years ago. In every instance, lower as well as upper, the gums showed little additional absorption, and were hard and healthy. I am constantly investigating mouths for the purpose of witnessing the relative effects of rubber and metal plates, and am more and more impressed with the great injury being done to mouths in this way. And it is really a serious matter to the individuals who are doomed to wear artificial teeth the rest of their lives, for they cannot fail to appreciate the fact that the better and more permanent the condition of the gums, the better will they be enabled to successfully and comfortably use their teeth. And so as I have said, let your patients know the real facts in the case, and then if they choose the cheap base, *base as it is*, the worse is their own.

